# Sperm selection with hyaluronic acid improved live birth outcomes among older couples and was connected to sperm DNA quality, potentially affecting all treatment outcomes

**DOI:** 10.1093/humrep/deac058

**Published:** 2022-04-23

**Authors:** Robert West, Arri Coomarasamy, Lorraine Frew, Rachel Hutton, Jackson Kirkman-Brown, Martin Lawlor, Sheena Lewis, Riitta Partanen, Alex Payne-Dwyer, Claudia Román-Montañana, Forough Torabi, Sofia Tsagdi, David Miller

**Affiliations:** Leeds Institute of Health Sciences, University of Leeds, Leeds, UK; Centre for Human Reproductive Science, University of Birmingham, Birmingham Women’s Fertility Centre, Birmingham Women’s NHS Foundation Trust, Birmingham, UK; Centre for Human Reproductive Science, University of Birmingham, Birmingham Women’s Fertility Centre, Birmingham Women’s NHS Foundation Trust, Birmingham, UK; Queen’s University Belfast, Centre for Public Health, Royal Groups of Hospitals, Belfast, UK; Centre for Human Reproductive Science, University of Birmingham, Birmingham Women’s Fertility Centre, Birmingham Women’s NHS Foundation Trust, Birmingham, UK; Queen’s University Belfast, Centre for Public Health, Royal Groups of Hospitals, Belfast, UK; Queen’s University Belfast, Centre for Public Health, Royal Groups of Hospitals, Belfast, UK; Department of Discovery and Translational Science, Leeds Institute of Cardiovascular and Metabolic Medicine (LICAMM), University of Leeds, Leeds, UK; Department of Discovery and Translational Science, Leeds Institute of Cardiovascular and Metabolic Medicine (LICAMM), University of Leeds, Leeds, UK; Centre for Human Reproductive Science, University of Birmingham, Birmingham Women’s Fertility Centre, Birmingham Women’s NHS Foundation Trust, Birmingham, UK; Department of Discovery and Translational Science, Leeds Institute of Cardiovascular and Metabolic Medicine (LICAMM), University of Leeds, Leeds, UK; Centre for Human Reproductive Science, University of Birmingham, Birmingham Women’s Fertility Centre, Birmingham Women’s NHS Foundation Trust, Birmingham, UK; Department of Discovery and Translational Science, Leeds Institute of Cardiovascular and Metabolic Medicine (LICAMM), University of Leeds, Leeds, UK

**Keywords:** hyaluronic acid, sperm selection, sperm function, sperm quality, IVF/ICSI outcome, clinical trial, mechanisms, defective sperm, sperm DNA, DNA quality

## Abstract

**STUDY QUESTION:**

What effects did treatment using hyaluronic acid (HA) binding/selection prior to ICSI have on clinical outcomes in the Hyaluronic Acid Binding sperm Selection (HABSelect) clinical trial?

**SUMMARY ANSWER:**

Older women randomized to the trial’s experimental arm (selection of sperm bound to immobilized (solid-state) HA) had the same live birth rates as younger women, most likely a result of better avoidance of sperm with damaged DNA.

**WHAT IS KNOWN ALREADY:**

Recent randomized controlled trials (RCTs) investigating the efficacy of HA-based sperm selection prior to ICSI, including HABSelect, have consistently reported reductions in the numbers of miscarriages among couples randomized to the intervention, suggesting a pathological sperm-mediated factor mitigated by prior HA-binding/selection. The mechanism of that protection is unknown.

**STUDY DESIGN, SIZE, DURATION:**

The original HABSelect Phase 3 RCT ran from 2014 to 2017 and included 2752 couples from whom sperm samples used in control (ICSI) and intervention (Physiological IntraCytoplasmic Sperm Injection; PICSI) arms of the trial were stored frozen for later assessment of DNA quality (DNAq). The trial overlapped with its mechanistic arm, running from 2016 to 2018.

**PARTICIPANTS/MATERIALS, SETTING, METHODS:**

As miscarriage reduction was a significant secondary outcome of the trial, samples (n* *=* *1247) selected for the mechanistic analysis were deliberately enriched for miscarriage outcomes (n = 92 or 7.4%) from a total of 154 miscarriages (5.6%) among all (n* *=* *2752) couples randomized by stratified random sampling. Values from fresh semen samples for sperm concentration (mml), percentage forward progressive motility and percentage HA-binding score (HBS) were obtained before being processed by differential density gradient centrifugation or (rarely) by swim-up on the day of treatment. Surplus sperm pellets were recovered, aliquoted and cryopreserved for later analysis of DNAq using slide-based Comet, TUNEL, acridine orange (AO) and the sperm chromatin dispersion (SCD) assays. Following their classification into normal and abnormal sample subcategories based on reference values for sperm concentration and motility, relationships with HBS and DNAq were examined by Spearman correlation, Student’s *t*-tests, Mann Whitney U tests, and logistic regression (univariable and multivariable). Parsimonious selection enabled the development of models for exploring and explaining data trends. Potential differences in future cumulative pregnancy rates relating to embryo quality were also explored.

**MAIN RESULTS AND THE ROLE OF CHANCE:**

Results from the 1247 sperm samples assayed for HBS and/or DNAq, generated data that were considered in relation to standard physiological measures of (sperm) vitality and to treatment outcomes. All measures of HBS and DNAq discriminated normal from abnormal sperm samples (*P* < 0.001). SCD correlated negatively with the Comet (*r* = −0.165; *P* < 0.001) and TUNEL assays (*r* = −0.200; *P* < 0.001). HBS correlated negatively with AO (*r* = −0.211; *P* < 0.001), Comet (*r* = −0.127; *P* < 0.001) and TUNEL (*r* = −0.214; *P* < 0.001) and positively with SCD (*r* = 0.255; *P* < 0.001). A model for predicting live birth (and miscarriage) rates included treatment allocation (odds ratio: OR 2.167, 95% CI 1.084–4.464, *P* = 0.031), female age (OR 0.301, 95% CI 0.133–0.761, *P* = 0.013, per decade) and the AO assay (OR 0.79, 95% CI 0.60–1. 02.761, *P* = 0.073, per 10 points rise). A model predicting the expected rate of biochemical pregnancy included male age (OR 0.464, 95% CI 0.314–0.674, *P* < 0.001, per decade) and the SCD assay (OR 1.04, 95% CI 1.007–1.075, *P* = 0.018, per 10 point rise). A model for conversion from biochemical to clinical pregnancy did not retain any significant patient or assay variables. A model for post-injection fertilization rates included treatment allocation (OR 0.83, 95% CI 0.75–0.91, *P* < 0.001) and the Comet assay (OR 0.950, 95% CI 0.91–1.00, *P* = 0.041).

**LIMITATIONS, REASONS FOR CAUTION:**

HABSelect was a prospective RCT and the mechanistic study group was drawn from its recruitment cohort for retrospective analysis, without the full benefit of randomization. The clinical and mechanistic aspects of the study were mutually exclusive in that measures of DNAq were obtained from residual samples and not from HA-selected versus unselected sperm. Models for fitting mechanistic with baseline and other clinical data were developed to compensate for variable DNAq data quality. HABSelect used a solid-state version of PICSI and we did not assess the efficacy of any liquid-state alternatives. PICSI reduced fertilization rates and did not improve the outlook for cumulative pregnancy rates.

**WIDER IMPLICATIONS OF THE FINDINGS:**

Notwithstanding the interventional effect on fertilization rates and possibly blastocyst formation (neither of which influenced pregnancy rates), poor sperm DNAq, reflected by lower HBS, probably contributed to the depression of all gestational outcomes including live births, in the HABSelect trial. The interventional avoidance of defective sperm is the best explanation for the equalization in live birth rates among older couples randomized to the trial’s PICSI arm. As patients going forward for assisted conception cycles globally in future are likely to be dominated by an older demographic, HA-based selection of sperm for ICSI could be considered as part of their treatment plan.

**STUDY FUNDING/COMPETING INTEREST(S):**

The study was supported by the National Institute for Health Research (NIHR) EME (Efficacy and Mechanism Evaluation)-11-14-34. National Research Ethics Service approval 11/06/2013: 13/YH/0162. S.L. is CEO of ExamenLab Ltd (company number NI605309).

**TRIAL REGISTRATION NUMBER:**

ISRCTN99214271.

## Introduction

Sperm DNA integrity, henceforth referred to as DNA quality (DNAq), is essential for generating viable pregnancies with strong evidence that lower DNAq compromises IVF success rates (Cissen *et al.*, 2016; Zidi-Jrah *et al.*, 2016; Simon *et al.*, 2017). With ICSI, the sperm is injected directly into the egg, bypassing many of the natural barriers that would normally prevent the entry of abnormal sperm. The relationship between DNAq and treatment outcome in ICSI is less clear, although miscarriage risk is elevated among couples where male partners have sperm with abnormally low DNAq (Robinson *et al.*, 2012; Osman *et al.*, 2015; Bach and Schlegel, 2016). These studies also suggest that there is an increased risk of miscarriage associated with the use of sperm from raw, unprocessed semen containing mixed cell populations compared with processed samples that are substantially cleared of poorer quality sperm, and Haddock *et al.* (2021) reported similar sperm DNAq values associated with miscarriage following either natural conception or assisted conception (Robinson *et al.*, 2012; Zhao *et al.*, 2014; Coughlan *et al.*, 2015; Cissen *et al.*, 2016; Haddock *et al.*, 2021).

Measuring sperm DNAq, which for the purpose of this report is defined as any structural aspect of sperm chromatin that can compromise sperm function if disrupted, is pivotal to our understanding of male infertility and its impact on ART outcomes. The connection between DNAq and reproductive success is indisputable, but there is no overall consensus on the relative merits of the various assays available to measure it (Robinson *et al.*, 2012; Zhao *et al.*, 2014). Five such assays are commonly used in Andrology settings with both slide-based and flow-cytometric variants available. Owing to convenience, cost and often sample limitations, slide-based assays including TUNEL, Comet, sperm chromatin dispersion (SCD) and acridine orange (AO) staining are popular (Donnelly *et al.*, 1999a,b; Fernandez *et al.*, 2003; De Sanctis *et al.*, 2008). These assays might reasonably be expected to show similar qualitative and quantitative behaviours in their capacity to detect anomalies in sperm DNAq (Chohan *et al.*, 2006; Ribas-Maynou *et al.*, 2014; Simon *et al.*, 2014). There is no agreed consensus or guidance, however, covering the relative merits or demerits of each one. There are also other factors of sperm DNA which may influence outcomes not measured by these assays, for example, telomere length, (Lafuente *et al.*, 2018), and DNA ploidy (Ovari *et al.*, 2010).

Sperm that bind to hyaluronic acid (HA), a major component of the extracellular matrix surrounding the oocyte–cumulus complex (Dandekar *et al.*, 1992), are reported to be more mature, have better DNAq, better DNA compaction and less residual cytoplasm (Huszar *et al.*, 2003; Parmegiani *et al.*, 2010; Mokanszki *et al.*, 2014; Rashki Ghaleno *et al.*, 2016). A sample’s HA-binding score (HBS) is usually reported as the percentage of sperm adhering to an immobilized and hence solid-state, HA-coated surface and depends on sperm concentration and motility in the ejaculates concerned (Mokanszki *et al.*, 2014; Rashki Ghaleno *et al.*, 2016). Several studies reporting the correspondence between HBS and standard measures of sperm function based on World Health Organization (WHO) criteria, suggest that men with abnormally low HBS are generally sub-fertile and therefore more likely to experience difficulty having offspring (Tarozzi *et al.*, 2009; Mokanszki *et al.*, 2014). The confidence of this assumption is such that others have suggested using HBS to help direct decision-making in the treatment of male infertility (Worrilow *et al.*, 2012; Mokanszki *et al.*, 2014; Michailidou-Ahmed *et al.*, 2016; Kirkman-Brown *et al.*, 2019).

Several studies have also evaluated the efficacy of HA-selected sperm in ART treatment cycles, with only the lowering of miscarriage rates being a common feature (Worrilow *et al.*, 2012; Mokanszki *et al.*, 2014; Erberelli *et al.*, 2017; Lepine *et al.*, 2019; Miller *et al.*, 2019). These reports have been less consistent with other outcome measures, including the establishment of biochemical and clinical pregnancy, and it is currently unclear if HA-selected sperm give rise to better quality embryos (Choe *et al.*, 2012; Parmegiani *et al.*, 2012; Lepine *et al.*, 2019) or if it helps to increase clinical or live birth rates (Nijs *et al.*, 2010; Choe *et al.*, 2012; Worrilow *et al.*, 2012; Mokanszki *et al.*, 2014; Beck-Fruchter *et al.*, 2016).

The Hyaluronic Acid Binding sperm Selection (HABSelect) trial was a blinded and randomized controlled trial (RCT) that ran from 2014 to 2018 in 16 major UK clinical treatment centres and tested the efficacy of HA-based sperm selection using a solid-state Physiological IntraCytoplasmic Sperm Injection (PICSI) platform approved by the Medical Health Regulatory Agency (MHRA). The study reported significantly reduced miscarriage rates in its PICSI arm (Miller *et al.*, 2019) and there were no other significantly different clinical outcomes. Unlike all previous studies, however, HABSelect included an effort to investigate and provide some mechanistic linkage between the general quality of the sperm used in the trial, with particular reference to HBS, DNAq and the trial’s clinical outcomes. The use of multiple assays of DNAq across many of the same samples made the HABSelect dataset ideally suited for this purpose.

At the time of publication (Miller *et al.*, 2019), it was argued that the significant impact of PICSI on miscarriage avoidance in the HABSelect clinical trial could have been a chance event. Here, we report updated evidence that lower HBS and DNAq were associated with poorer sperm quality that compromised treatment outcomes throughout the gestational timeline. We are also more confident that HA-based selection mitigated the deleterious effects of damaged sperm DNA on final treatment outcomes, particularly among older women. We also consider the relevance and usefulness of HBS and DNAq measures in relation to standard semen analysis and to treatment outcomes.

## Materials and methods

### Ethics

HABSelect was a parallel arm, double-blinded RCT aimed at testing the efficacy of HA-selection of sperm prior to ICSI (Physiological ICSI) for improving live birth outcomes. The trial used the UK’s MHRA approved solid-state HA-binding platform, PICSI for this purpose, (CooperSurgical, #BCT-PICSI-20, UK). The study was approved by the UK National Research Ethics Service (approval number 13/YH/0162). Secondary outcome measures included biochemical pregnancy, clinical pregnancy and miscarriage rates. A solid-state PICSI platform was chosen solely because of the tightly controlled technical standard of its manufacture and its ready availability. The full trial rationale, including a protocol summary with inclusion and exclusion criteria, are reported elsewhere (Witt, 2016; Kirkman-Brown *et al.*, 2019). The mechanistic analysis as described in the trial protocol was hypothesis generating and not testing. Its purpose was to explore relationships between clinical and experimental measures/outcomes. The study aimed to link measures of patient baseline data and sperm HBS, with sperm DNAq and embryo quality and the trial’s clinical outcomes. The mechanistic cohort (see below) was sampled from couples randomized for treatment allocation within the trial, making this an observational mechanistic study without the full benefit of randomization. The mechanistic laboratory teams were always blinded from patient data and were therefore unaware of related outcomes.

All couples recruited to the HABSelect RCT had read a detailed information sheet describing the trial and its goals and all semen samples were obtained after patients had given signed consent to their use in this scientific study.

### Sperm preparation and processing for storage

Semen samples were obtained on the day of treatment by masturbation into sterile containers. As we were interested in exploring the possibility that some miscarriages were male-mediated (Kirkman-Brown *et al.*, 2019) and as miscarriage was the only significant clinical outcome of the original HABSelect RCT, a miscarriage-enriched sample set was retrospectively selected for mechanistic analyses without those involved in DNAq assaying being aware of associated clinical outcomes. Sample volume (ml), sperm concentration (mml), forward progressive motility (%) and HBS (see below) were obtained on the day of treatment and before semen samples were processed by differential density gradient centrifugation, or occasionally by swim-up, using standard methods (WHO) (Cooper *et al.*, 2010). Sample physiological baseline parameters are presented in Table I.

**Table I:** Baseline statistics and other relevant parameters stratified (*) by sample classification.

	Normal	Abnormal	*P*-value
Male baseline and other relevant parameters	N = 399	N = 816	
Age, years (mean (SD))^$^	36.46 (5.47)	35.89 (5.56)	0.092
BMI, kg/m^2^ (mean (SD))^$^	27.54 (4.53)	26.84 (4.29)	0.075°
Median sperm conc., mml (IQR)^#^	42.4 (25.0, 67.4)	7.0 (20.8, 12.6)	<0.001**
Mean sperm conc., mml (SD)^$^	52.5 (±37.6)	13.1 (±25.1)	<0.001**
% median prog for mot (IQR)^#^	51.0 (42.0, 63.0)	33.0 (20.8, 50.0)	<0.001**
% mean prog for mot (SD)^$^	52.5 (±13.0)	35.5 (±19.8)	<0.001**
Median sample vol ml (IQR)^#^	2.5 (1.9, 3.4)	2.8 (2.0, 4.0)	<0.001**
Median HBS (IQR)^#^	87.5 (74.5, 93.0)	81 (55.0, 90.75)	<0.001**
Mean sample vol mL (SD)^$^	2.7 (±1.3)	3.1 (±1.5)	<0.001**
Smoker (%)^χ2^			
No	379 (95.9)	764 (94.6)	0.367
Yes	16 (4.1)	44 (5.4)	
Cig cons (mean (SD))^$^	10.53 (5.57)	8.21 (4.38)	0.107
Drinker (%)^χ2^			
No	144 (37.9)	303 (39.6)	0.632
Yes	236 (62.1)	463 (60.4)	
Alcohol cons units/week (mean (SD))^$^	7.86 (6.15)	7.93 (7.09)	0.898
Recreational drug (%)^χ2^			
No	364 (99.7)	764 (99.9)	1
Yes	1 (0.3)	1 (0.1)	
Allocation (%)^χ2^			
ICSI	201 (50.4)	403 (49.4)	0.793
PICSI	198 (49.6)	413 (50.6)	
Outcome (%)^χ2^			
No pregnancy	207 (51.9)	431 (52.8)	0.695
Miscarriage	32 (8.0)	57 (7.0)	
Pre-term	11 (2.8)	31 (3.8)	
Term birth	126 (31.6)	240 (29.4)	

Data from the 1215 samples with selected male baseline measures for couples in the mechanistic cohort are here shown stratified by semen sample classification (normal or abnormal) according to WHO 2010 lower reference values. Abnormal includes any freshly ejaculated sample on the day of treatment with sperm conc ≤15 mml or forward progressive motility ≤31% or both.

Potential differences between category values were checked using *t* tests (^$^), Mann–Whitney *U* tests (^#^) and Chi-square (^χ2^) tests. As expected, physiological aspects of semen quality differed between the two classes but there were no other differences. Clinical treatment outcomes did not differ and are shown for information only. **Indicates very highly significant *P*-value (p < 0.001).

% mean/median prog for mot, % mean/median progressive forward motility; Alcohol cons, alcohol consumption units/week; Cig cons, number of cigarettes/cigars consumed/week; HBS, hyaluronic acid binding score; IQR, interquartile range; Mml, millions of sperm per ml; SD, standard deviation from the mean.

### Assaying for sample quality

**Figure 1.**
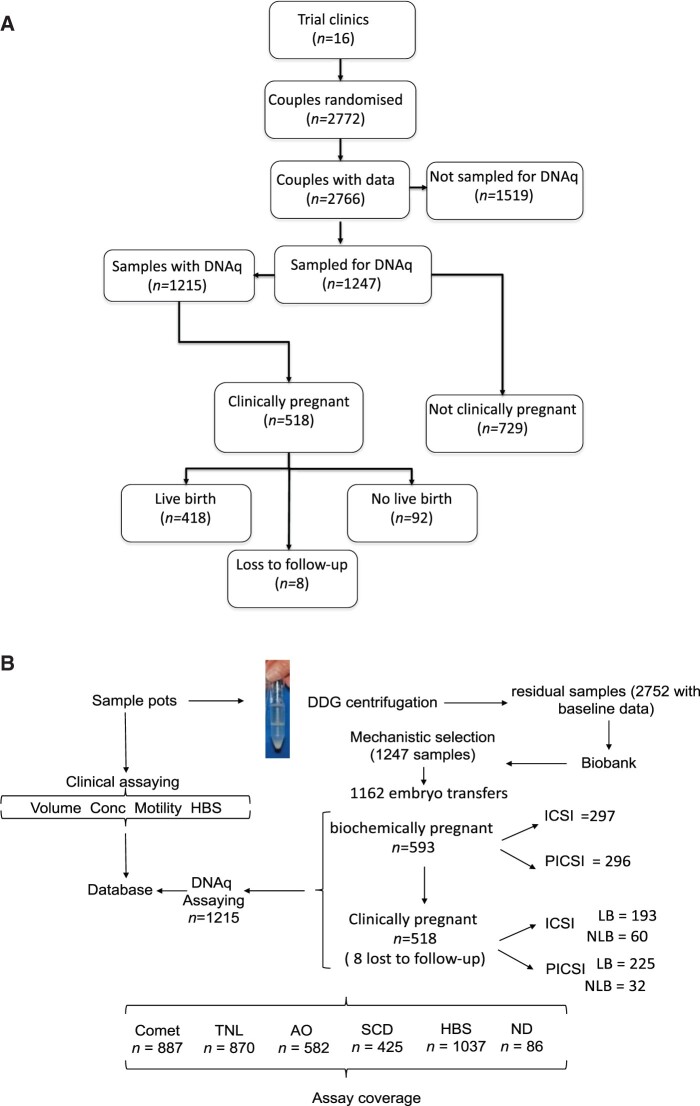
**CONSORT chart for the mechanistic cohort and sample processing pipeline.** Of the 2772 couples randomized in the Hyaluronic Acid Binding sperm Selection (HABSelect) clinical trial (**A**), 1247 comprised the mechanistic cohort although owing to clinical, technical and time constraints, 1215 were finally sampled for DNA quality (DNAq). Two samples were associated with couples without eggs and eight samples were associated with clinical pregnancies lost to follow-up. The sample processing pipeline (**B**) shows the relationships between sample acquisition for the full trial cohort (n = 2752), those samples selected for processing (n = 1245) and covering samples associated with embryo transfers (n = 1162). See Materials and methods and Results sections for full details. DDG, differential density gradient; ND, no data; PICSI, physiological intracytoplasmic sperm injection; TNL, TUNEL assay.

Following HBS scoring (see below), patients’ residual processed sperm were centrifuged (×500*g*) for 5 min in sperm wash buffer (SWB, CooperSurgical, UK) and resuspended in 0.5 ml SWB prior to the slow addition (0.7:1) of cryoprotectant (SpermFreeze™, Vitrolife, Sweden) according to the supplier’s instructions. Following careful incubation and mixing on ice, the samples were aliquoted (4 × 250 µl) and transferred to the vapour phase of liquid nitrogen for 20 min at −186°C prior to liquid storage at −196°C. Samples were shipped to and from the central biostore (Birmingham Biobank) and to all three mechanistic laboratories on solid CO_2_ (−80°C). As HABselect was testing the efficacy of an HA-selection process in ICSI treatments, HBS were obtained using the Hydak slide (Sterling-Cooper, UK) according to the supplier’s instructions with results expressed as percentage sperm tethered to the HA substrate (Torabi *et al.*, 2017). Briefly, 1 × 10^6^ sperm in 10 μl of SWB were placed onto the assay chamber and incubated at room temperature for 15 min. Spermatozoa with HA receptors bind to the coated slide while those lacking the receptors can continue to move around freely. Immotile cells are ignored. Percentage values for HA-bound spermatozoa per sample were calculated as (bound motile/total motile) × 100. To assess corresponding DNAq, stored processed sample aliquots were thawed rapidly at 37°C and prepared for one or more of the assays of DNAq essentially following the published protocols for AO staining (Tejada *et al.*, 1984; Yagci *et al.*, 2010), the alkaline Comet assay (Donnelly *et al.*, 1999a,b), TUNEL assay (De Sanctis *et al.*, 2008) and SCD assay (Fernandez *et al.*, 2003). A consort chart for trial sample acquisition and a flow chart of the mechanistic processing pipeline are shown in Fig. 1.

Brief descriptions of the DNAq protocols based on the trial’s standard operating procedures are presented below, while the more specialist bespoke image processing and quantification aspects of staining variables applied for the AO assay are provided in Supplementary Data. For all assay procedures (except HBS and SCD), after rapid thawing of samples at 37°C and thereafter keeping on ice, sperm were washed free of cryo-protectant by re-suspension in an equal volume of PBS, centrifuging for 500*g* for 5 min, removing supernatants and repeating twice over with PBS at 4°C. Volumes were adjusted by dilution or concentration by centrifugation (500*g*) to permit application of approximately 200 000–500 000 sperm in 10–20 µl PBS (unless otherwise stated) on poly-l-lysine coated slides (Thermo Scientific, UK) and allowed to dry overnight.

#### Acridine orange

For the AO assay, slides were rinsed in distilled water and transferred to 0.1M HCl for 30 s followed by 0.1M NaOH for 30 s. Sperm were then fixed in modified Carnoy’s solution containing methanol (M/4056/17; Fisher Scientific, Loughborough, UK)/glacial acetic acid (A/0400: PB17; Fisher Scientific) at a 9:1 ratio (Yagci *et al.*, 2010) for 2 h at room temp, rinsed with distilled water and air dried for at least 60 min. Samples were stained under subdued lighting with freshly prepared AO solutions (# 24603; Polysciences Inc., Hirschberg an der Bergstraße, Germany, 12 µg/ml in distilled water) for 5 min at room temperature. Slides were rinsed free of AO with three changes of distilled water for 5 min each, with constant stirring, and allowed to air dry before applying cover slips with DPX mountant (without antifade; # 44581, Sigma-Aldrich, Gillingham, UK). A Zeiss Axioplan II epifluorescence microscope (Boston Industries, Walpole, MA, USA) fitted with an ORCA CMOS camera (Hamamatsu, Welwyn Garden City, UK) was used to capture images (×400) with SmartCapture 3 software (DSKU Ltd, Cambridge) on an iMac (Apple, UK) running macOS (El Capitan). All digitized quanta were processed through ImageJ to threshold, segment and integrate signals from individual sperm, which were then adjusted to account for exposure times and image field backgrounds, prior to final data export and calculation of the % sperm DNA fragmentation (DFI = 100/(1 + green/red ratio)). See Supplementary Data for full details.

#### Sperm chromatin dispersion

For the SCD assay, the commercial Halosperm kit (Microm UK) was used with samples processed according to the supplier’s instructions. In brief, 15–25 µl thawed sample aliquots were mixed with prepared low melting point (57°C) agarose held at 37°C and applied to the slides, which were then cooled at 4°C for at least 5 min. Slides were then flooded with denaturing solution (supplier’s protocol) and incubated for 7 min at room temperature. Slides were then immersed in lysis solution (supplier’s protocol) for 25 min followed by submersion in distilled water for 5 min. Slides were then sequentially processed for 2 min each through solutions of 70%, 90% and 100% ethanol and allowed to air dry. Slides were flooded with a 10% (v/v); Giemsa stain (GS500, Sigma Aldrich, Gillingham, UK) and washed gently in distilled water to remove excess stain. Bright-field images (16 bit) were captured using a Basler Ace camera mounted on a Zeiss Primostar microscope (×100). Halo area data were acquired using SCA’s custom DNA module (Microm Ltd, Bicester, UK). Following slide calibration, individual sperm halo areas reported as pixels^2^ were exported on Comma Separated Value (CVS) delimited spreadsheets for further analysis.

#### TUNEL

For the TUNEL assay, the *in situ* cell death detection kit was used according to the manufacturer’s instructions (Roche, UK; De Sanctis *et al.*, 2008). Briefly, prepared samples dried on to slides were incubated in 2 mM dithiothreitol (DTT, D9163-5G, Sigma, Gillingham, UK) for 45 min at room temperature. Slides were then washed in PBS for 5 min and fixed in 4% w/v paraformaldehyde (158127, Sigma, Gillingham, UK), in PBS (P4417, Sigma, Gillingham, UK) for 15 min on ice followed by washing with PBS (3 × 5 min each). Slides were transferred to a permeabilizing solution (10 mg sodium citrate, S4641, Sigma, Gillingham, UK); 10 μl Triton x-100 (X100-100, Sigma, Gillingham, UK) in 10 ml distilled water, for 2 min on ice. Slides were then washed in PBS (2 × 5 min each) and allowed to air dry. TUNEL labelling solution was prepared according to the manufacturer’s instructions and 25 μl aliquots applied to slides as required. Following the addition of coverslips, slides were incubated for 60 min at room temperature in subdued lighting. Images (×600) were obtained on an Olympus BX61 microscope (Cambridge, MA, USA) fitted with epifluorescence optics and a Quantum 512SC camera (Photometrics, London, UK). Results are reported as % sperm with fluorescing heads among at least 200 counted.

#### Alkaline Comet

For the alkaline Comet assay (Donnelly *et al.* 1999a,b; Haddock *et al.* 2021), aliquots of native semen were adjusted using PBS to give a sperm concentration of 2 × 10^6^ mL^−1^ and embedded in agarose. Embedded cells were then subjected to membrane lysis, protamine and histone removal, electrophoresis, SYBR Gold staining and Comet scoring (Komet 7.0, Andor Technologies, Belfast, UK) with analysis of 50 sperm cells per slide, in duplicate. All steps were carried out in a temperature and humidity-controlled environment to prevent induction of DNA damage during processing. Previous studies have reported an intra-assay coefficient of variation of 6% for this assay (Donnelly *et al.*, 2000; Agbaje *et al.*, 2007).

### Data sampling and statistical analysis

As miscarriage reduction was the only significant clinical outcome from the HABSelect trial, the mechanistic cohort (n = 1247), through stratified random sampling, included a higher proportion of miscarriage outcomes than was the case for the full trial cohort. This detail was blinded to those undertaking the DNAq assays. The former included 92/1247 (7.4%) miscarriages from a total of 154/2752 (5.6%) miscarriages among couples randomized in the full clinical trial (a 2.1% enrichment). Relationships between HBS and DNAq with embryo quality and clinical outcomes were explored indirectly by aggregating the original data into 10-year intervals for patient age and 10-point differences for measures of sperm HBS and DNAq. Data were then analysed by Student’s *t*-tests, Mann–Whitney *U* tests, **χ**^2^ tests and by Spearman rank correlation to compare baseline and other related data and by univariable and multivariable logistic regression followed by parsimonious filtering to generate models for predicting clinical outcomes. Modelling was intended to improve clarity for emphasizing trends in the data, otherwise hidden by noise. All statistical analyses were undertaken using R statistical software, version 4.0.2 (R Core Team, 2020). Statistical significance was set at the 5% level. These analyses in turn provided useful hypothesis-generating information linking assay data with clinical outcomes.

## Results

### Processing pipelines and relationships between patient baseline characteristics, standard measures of semen quality and assay outcomes (HBS and DNAq)

Figure 1 shows the clinical progression (Fig. 1A) for couples (n = 2772) randomized for treatment and then following mechanistic selection (see Materials and methods for selection criteria) entering the mechanistic processing pipeline (Fig. 1B; n = 1247). Fertility clinics were responsible for obtaining all baseline measures on fresh semen including sample volume, sperm concentration, forward progressive motility and HBS on the day of treatment (Table I). All other measures were obtained retrospectively from the associated mechanistic laboratories following thawing of frozen-stored samples. Of 1247 selected processed frozen samples making up the mechanistic cohort with full baseline data, two couples had no eggs to fertilize, 1215 couples had measures of sperm DNAq of which 1162 had embryo transfers. Treatment outcomes are shown where appropriate.

Baseline mechanistic patient and assay data are summarized in Table I according to sample quality, classified as abnormal if the original fresh semen sample obtained on the day of treatment had a sperm concentration <15 mml or forward progressive motility <32% or both (Cooper *et al.*, 2010 and WHO 2010 lower reference values). This led to classification of the 1215 available samples with HBS and DNAq data into normal (n* *=* *399) and abnormal (n = 816) subgroups, as shown. Sample classification not unexpectedly led to marked differences in mean and median values for both sperm concentration and progressive motility. No other differences were noted for baseline measures in either class and the equipoise for treatment allocation was preserved. Subsequent clinical outcomes, considered here by a portfolio test (*P* = 0.695), did not differ between classes. The same baseline parameters involving the full mechanistic cohort (n* *=* *1247) less two couples with no eggs are also summarized according to treatment allocation (Table II). With the exception of fertilization rates, there were no significant differences in patient baseline characteristics, clinical outcomes or measures of sperm HBS and DNAq stratified by treatment allocation, although live birth rates (here reported according to all couples in the mechanistic cohort) were slightly elevated in the PICSI cohort.

**Table II:** Baseline statistics and other relevant parameters stratified by treatment allocation.

Patient baseline and other relevant parameters	ICSI	PICSI	*P*-value
**Male characteristics (n)**	619	626	
Age (mean (SD))^$^	35.94 (5.32)	36.22 (5.75)	0.373
BMI (mean (SD))^$^	26.90 (4.34)	27.22 (4.37)	0.374
Alcohol cons units/week (median [IQR])^#^	6.00 [3.00, 10.00]	6.00 [3.00, 10.00]	0.938
Cig cons (mean (SD))^$^	0.05 (0.23)	0.05 (0.21)	0.636
Mean sperm conc., mml (SD)^$^	13.00 [4.55, 36.50]	12.80 [5.00, 33.62]	0.944
Median sperm conc., mml (IQR)^#^	18.00 [6.85, 40.00]	17.75 [5.60, 39.00]	0.786
Sperm conc., mml category^χ2^			
<15 × 10^6^	329 (53.2)	330 (52.7)	
≥15 × 10^6^	282 (45.6)	286 (45.7)	0.899
Mean sample vol, ml (SD)^$^	2.99 (1.59)	2.93 (1.42)	0.507
% mean prog for mot (SD)^$^	42.48 (20.15)	40.40 (18.80)	0.067
% median prog for mot (IQR)^#^	72.34 (25.21)	72.12 (24.98)	0.887
HBS (mean (SD))^$^	74.99 (23.88)	73.08 (24.78)	0.204
**Female characteristics (n)**			
Age (mean (SD))^$^	33.83 (4.19)	33.74 (4.34)	0.72
BMI (mean (SD))^$^	24.25 (3.55)	24.51 (3.49)	0.193
FSH (miU/ml) (mean (SD))^$^	7.12 (2.27)	7.00 (2.02)	0.421
AMH (pmol/l) (mean (SD))^$^	21.53 (18.15)	21.89 (17.82)	0.799
**Treatment outcomes**			
Fertilization rate (mean (SD))^$^	0.71 (0.22)	0.68 (0.24)	0.007**
PNZ (mean (SD))^$^	6.22 (4.07)	6.02 (4.04)	0.397
Biochemical pregnancy (mean (SD))^$^	0.48 (0.50)	0.47 (0.50)	0.893
Clinical pregnancy (mean (SD))^$^	0.41 (0.49)	0.42 (0.49)	0.912
Live births (%)^χ2^	193 (31.1)	225 (36.0)	0.078°
No live birth (%)^χ2^	427 (68.9)	400 (64.0)	
**Assays (mean (SD))** ^$^			
AO frag	65.31 (13.70)	65.23 (14.97)	0.942
Comet frag	19.22 (9.96)	18.63 (9.11)	0.357
SCD (halo area) pixel^a^	173.57 (63.01)	172.71 (63.36)	0.888
TUNEL frag	12.33 (15.01)	12.32 (14.81)	0.993
HBS	74.99 (23.88)	73.08 (24.78)	0.204

Data from 1245 samples comprising the full mechanistic cohort (n* *=* *1247) less two couples with no eggs are shown stratified by treatment allocation for PICSI and ICSI.

Potential differences between category values were checked using *t* tests (^$^), Mann–Whitney *U* tests (^#^) and Chi-square (^χ2^) tests.

The table shows that all patient and sample characteristics that should have been independent of allocation did not differ between the subgroups. Although the proportions of normal and abnormal samples in each subgroup were identical, live birth outcomes were weakly influenced by allocation choice (°).

Assays of DNAq (AO, Comet, SCD and TUNEL) reported as % sperm showing DNA fragmentation frag except SCD which measures halo area in pixel^a^. HBS reported as % motile sperm binding to the Hydak slide.

% mean/median prog for mot, mean/median % progressive forward motility; alcohol cons units/week, alcohol consumption units/week; AMH (pmol/l), anti-Mullerian hormone picomoles per litre; AO, acridine orange; cig cons, cigarette/cigar consumption/week; DOA, day of assessment; FSH (mIU/ml), FSH, milli international units per millilitre; HBS, hyaluronan binding score; IQR, interquartile range; PNZ, pronucleate zygote; SCD, sperm chromatin dispersion; TUNEL, terminal deoxynucleotidyl transferase dUTP nick end-labelling.

**Figure 2.**
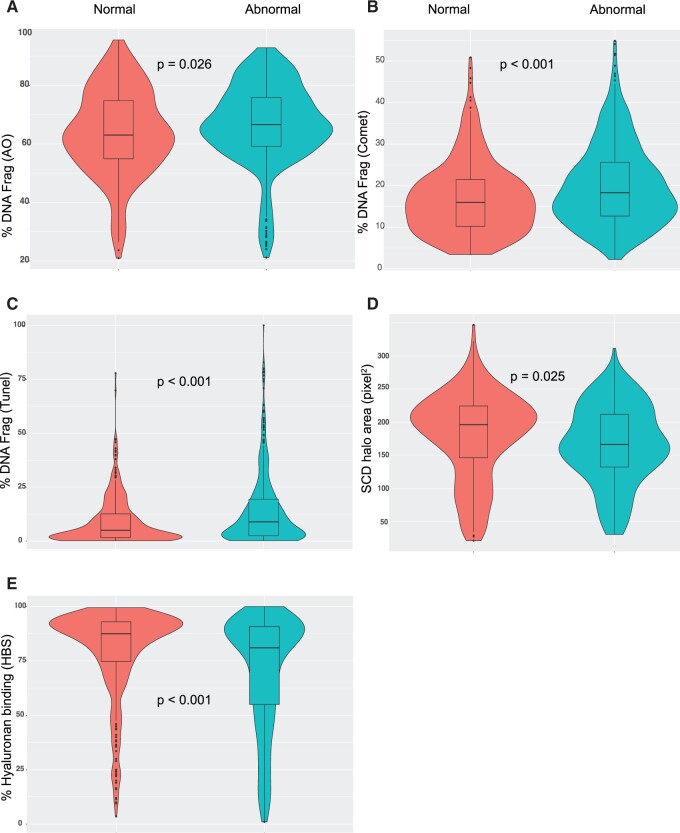
**Comparing HBS and DNAq measures from normal and abnormal sample subgroups.** By considering the World Health Organization 2010 lower reference limits for sperm concentration (15 mml) and forward progressive motility (31%), samples were classified into normal (n = 399) and abnormal (n = 816) if they were at or below these limits for either or both measures. Full details of the semen and other parameters of the subgroups are shown in Table I. The violin plots show the quartiles (boxes), minima and maxima (whiskers) and extreme outliers indicated by filled circles for % sperm with DNA fragmentation measured by Acridine orange (AO) (**A**), Comet (**B**), TUNEL (**C**); halo area (pixels^2^) by sperm chromatin dispersion (SCD) (**D**) and with % binding to hyaluronic acid binding score (HBS) (**E**). Plots also show the distribution of the data generating these values, highlighting where the data are more (fatter) or less (leaner) densely distributed. The derivation of HBS and DNAq data is provided in Materials and methods and in Supplementary Data. All quartile and mean values from the plots are shown in Supplementary Table SI alongside significance values determined by Mann–Whitney *U* test.

Figure 2 shows the relationships among assay measures following the same sample classification criteria, presented as violin plots where boxes show the 25%, median and 75% quartile values with whiskers connecting the minima and maxima. Shading highlights the uneven spread across the data, with high degrees of skew throughout. Among all assays, data for HBS and TUNEL showed the greatest skew, where most samples returned HBS scores of >65% and <15% for sperm DNA fragmentation (SDF). Irrespective of data skew, all relationships between baseline semen parameters and HBS or DNAq were as expected. For example, the median value for SCD halo area in the normal class (196.4 pixel^2^) was significantly higher than the abnormal class (166.6 pixel^2^), while the Comet assay returned significantly lower median % fragmentation in the normal (16%) versus abnormal (18%) classes. All *P*-values for these relationships are shown. Median and mean values for HBS were statistically higher in the normal than abnormal classes. Supplementary Table SI includes all the main values plotted in Fig. 2.

Looking next at the relationships between measures of DNAq from a total of 4326 assays overall carried out on the 1215 available samples for this purpose (Supplementary Table SII), the coverage ranged from 195 (full coverage with all assays; none missing) to 86 without any coverage (all missing). The inter-assay correlation matrix (Spearman Rho) for all possible assay pairs with available data is shown in Table III. Considering DNAq assays alone, only the SCD showed weak but significant correlations with TUNEL (*r* = −0.200; *P* < 0.001) and Comet (*r* = −0.165; *P* = 0.001) and in the expected (negative) directions where (for example) larger SCD halos correspond to lower levels of DNA fragmentation. HBS correlated significantly with all measures of DNAq and in the expected slope direction.

**Table III:** Inter-assay correlations.

Assay	AO	Comet	TUNEL	SCD (halo)	HBS
*AO	1.000				
*Comet	0.049 (n = 517; *P* = 0.26)	1.000			
*TUNEL	0.037 (n = 495; *P* = 0.41)	0.054 (n = 728; *P* = 0.14)	1.000		
*SCD (halo)	0.085 (n = 250; *P* = 0.18)	**−0.165 (n = 374; *P* = 0.001)**	**−0.200, (n = 377; <0.001)**	1.000	
**HBS**	**−0.211 (n = 544; *P* < 0.001)**	**−0.127 (n = 836; <0.001)**	**−0.214 (n = 794; *P* < 0.001)**	**0.255 (n = 397; <0.001)**	1.000

Matrix of Spearman rank correlations (Rho) for pairwise comparisons across DNAq and HBS observations. The numbers of samples with available paired data are indicated (*n*) followed by the correlation and *P*-values (significant correlations shown in **bold**). Of the DNAq assays, only SCD showed significant correlations with Comet and TUNEL. HBS correlated with all DNAq assays. All relationships correlated in the expected (slope) direction.

AO, acridine orange; HBS, hyaluronan binding score; SCD, sperm chromatin dispersion; TUNEL, terminal deoxynucleotidyl transferase dUTP nick end-labelling.

* Assays of DNAq (AO, Comet, SCD and TUNEL) reported as % sperm showing DNA fragmentation (frag except SCD which measures halo area in pixel^2^).

HBS reported as % motile sperm binding to the Hydak slide.

### Interventional effects alongside sperm HBS and DNAq in relation to clinical outcomes

**Figure 3.**
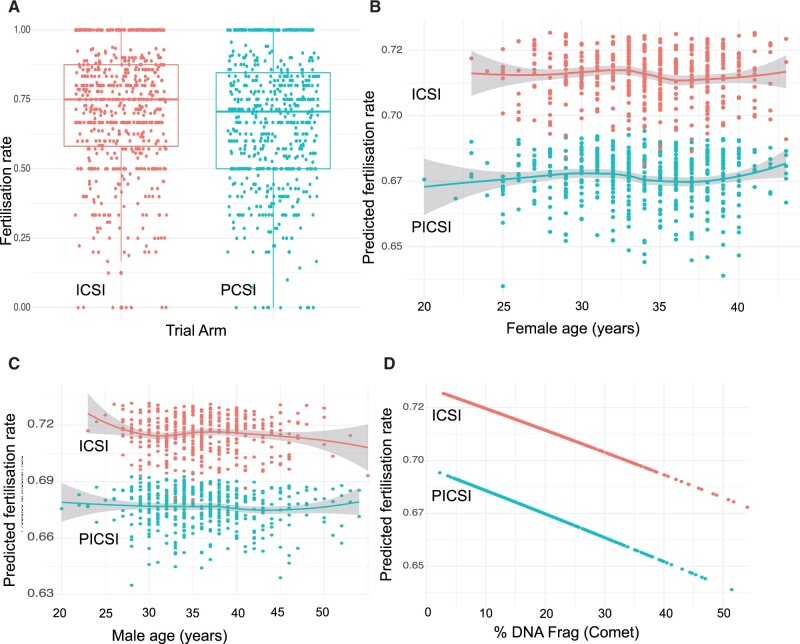
**Fertilization rates following ICSI or PICSI.** Baseline data are plotted according to treatment allocation (PICSI or ICSI) and showing quartiles, minima and maxima (**A**). Following data aggregation (into 10-year intervals for age and 10-scale points for HBS and DNAq), plots for the model predicting fertilization rates (0.00; no fertilization, 1.00; 100% fertilized) retained treatment allocation shown in relation to female (**B**) and male (**C**) age and the Comet assay (**D**). Note that increasing levels of DNA fragmentation were associated with lower predicted fertilization rates in both arms of the trial arm. Plots show moving average and surrounding 95% CI envelopes where appropriate. The absence of scatter in the Comet plot is because DNAq was the only variable, other than treatment allocation, with a significant impact on predicted fertilization rates. Odds ratios for fertilization rates are presented in Table IV. DNAq, DNA quality, HBS, hyaluronic acid binding score; PICSI, physiological intracytoplasmic sperm injection.

**Figure 4.**
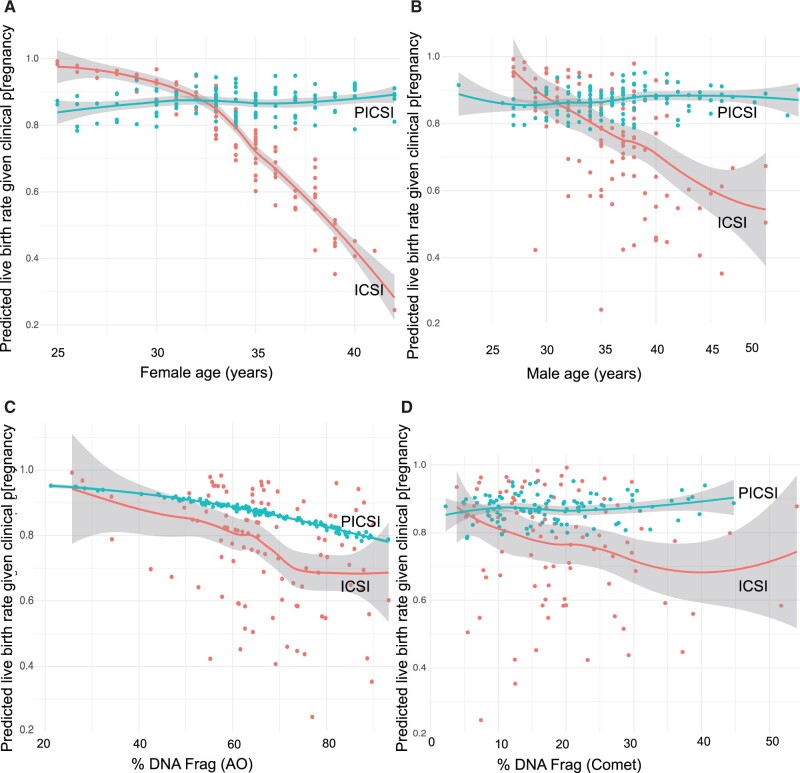
**Predicting live birth rates following ICSI or PICSI.** Following data aggregation as above, a model for predicting rates of live birth retained treatment allocation, as shown here, in relation to female (**A**) and male (**B**) age along with the AO assay (**C**). The Comet assay (**D**) is also shown because its predictive value by univariable analysis was close to that of AO. Plots show moving average and surrounding 95% CI envelopes with predicted live birth rates. Note the strong mitigating effect of PICSI treatment on falling births among older women, which is also responsible for the absence or reduction of scatter in the PICSI plots for DNAq. Scales for clinical pregnancies giving rise to live births are shown ranging from 20% (0.20) to 100% (1.00). Odds ratios for live birth are presented in Table IV. AO, acridine orange; DNAq, DNA quality; PICSI, physiological intracytoplasmic sperm injection.

**Figure 5.**
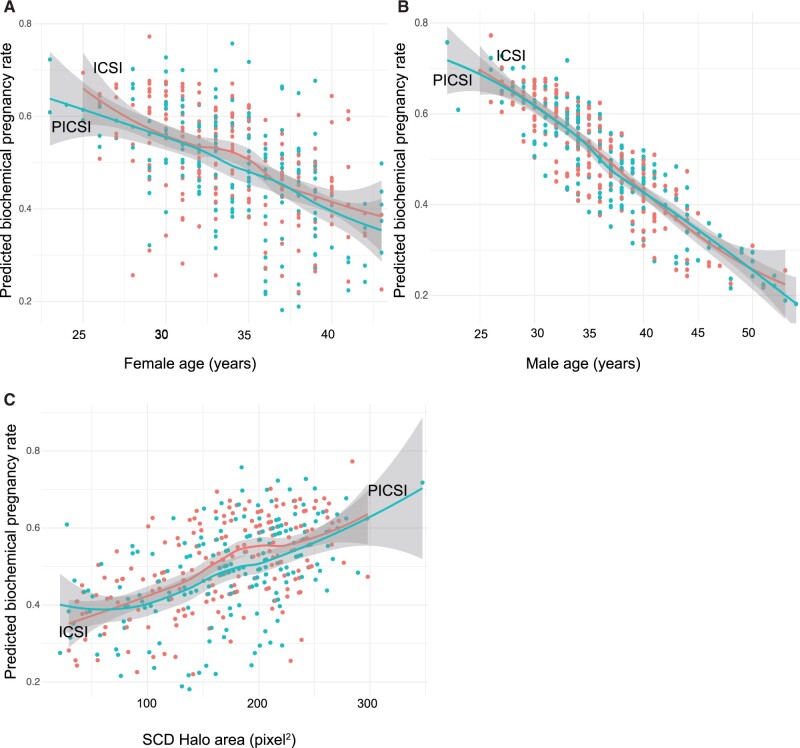
**Predicting biochemical pregnancy rates following ICSI or PICSI.** Following data aggregation as above, a model for predicting biochemical pregnancy rates is shown here in relation to female (**A**) and male (**B**) age. The model retained male age and the SCD assay presented as halo area in pixel^2^ units (**C**). Plots show moving average and surrounding 95% CI envelopes where appropriate. Note the absence of any treatment effect. Scales for embryo transfers generating biochemical pregnancies are shown ranging from 20% (0.20) to 100% (1.00). Odds ratios for biochemical pregnancy are presented in Table IV. SCD, sperm chromatin dispersion.

**Figure 6.**
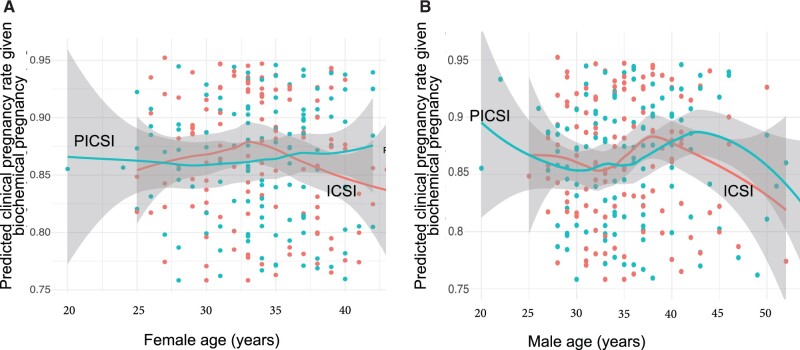
**Predicting rates of conversion from biochemical to clinical pregnancy following ICSI or PICSI.** The equivalent model for predicting successful conversion from biochemical to clinical pregnancy is plotted in relation to female (**A**) and male (**B**) age. Plots show moving average and surrounding 95% CI envelopes where appropriate. As indicated by unvariable regression, male age was predictive for conversion but only weakly so. Scales for conversion rates are shown ranging from 75% (0.75) to 95% (0.95). Odds ratios for clinical pregnancy are presented in Table IV. PICSI, physiological intracytoplasmic sperm injection.

We next explored the relationships between treatment allocation and measures of sample HBS and DNAq with clinical outcomes. Figures 3–6 show respective outputs from the models where variations in assay data and patient age predicting clinical outcomes generated the trend lines, CIs and the surrounding data scatter seen in all figures. In relation to treatment allocation, only fertilization rates (Fig. 3) and live birth/miscarriage outcomes (Fig. 4) differed significantly. Odds ratios (ORs) with CIs were calculated in relation to all clinical outcomes illustrated in these figures and are listed in Table IV. Fertilization rates were lower in the PICSI than ICSI cohorts (Fig. 3A; 68% versus 71%) and the reduction was independent of female (Fig. 3B) age, although a trend for slightly decreasing rates in older males (Fig. 3C) was also noted, restricted to the ICSI cohort. Regardless of treatment allocation, sperm DNA fragmentation (SDF) rates as measured by the Comet assay (Fig. 3D), was also predictive of fertilization, suggesting that DNAq factored in the success or otherwise of PNZ formation. Although the reduction in fertilization rates with PICSI had no effect on respective embryo transfer rates (see Discussion more details), we checked whether treatment allocation affected developing embryo quality and for any associated differences in assay measures (Supplementary Table SIII). We found slightly higher proportions of degenerate embryos (+1.58%) and fewer embryos destined for cryopreservation (−1.27%) in the ICSI arm, with neither difference reaching significance at the 5% level. There were also no differences in the numbers of transferred embryos in both arms of the trial. Hence, pre-transfer effects of PICSI would be unlikely to translate through to meaningful differences in future cumulative pregnancy rates.

**Table IV:** Models integrating sperm function assays with clinical outcomes by gestational progression.

	Outcome		0	1	OR (Uv 95% CI)	*P*-value	OR (Mv 95% CI)	*P*-value
	**fert (pnz)**							
Allocation (n)		ICSI (n = 619)	29%	71%				
Allocation (n)	Mean (SD)	ICSI (n = 619)	29%	71%				
		PICSI (n = 626)	32%	68%	0.837 (0.771–0.907)	*P* < 0.001	0.830 (0.754–0.913)	*P* < 0.001**
Female age^a^	Mean (SD)				0.910 (0.827–1.002)	*P* = 0.054*		
Male age^a^	Mean (SD)				0.929 (0.864–1.000)	*P* = 0.050*		
HBS^a^					1.022 (1.003–1.041)	*P* = 0.020*		
AO frag^a^	Mean (SD)				0.970 (0.931–1.011)	*P* = 0.152		
Comet frag^a^	Mean (SD)				0.954 (0.910–1.002)	*P* = 0.061	0.950 (0.906–0.998)	*P* = 0.041*
TUNEL frag^a^	Mean (SD)				0.965 (0.936–0.996)	*P* = 0.026*		
SCD halo area^a^	Mean (SD)				1.010 (1.000–1.021)	*P* = 0.055		

	**bioch preg**	MET	285 (47.9)	310 (52.1)				
			284 (50.1)	283 (49.9)	0.916 (0.728–1.153)	*P* = 0.456		
								
Allocation (%)		ICSI (n = 578)	281 (48.6)	297 (51.4)				
		PICSI (n = 584)	288 (49.3)	296 (50.7)	0.972 (0.773–1.224)	*P* = 0.812		
Female age^a^	Mean (SD)		3.4 (0.4)	3.3 (0.4)	0.608 (0.460–0.801)	*P* < 0.001**		
Male age^a^	Mean (SD)		3.7 (0.6)	3.5 (0.5)	0.625 (0.504–0.773)	*P* < 0.001**	0.464 (0.314–0.674)	*P* < 0.001**
HBS^a^	Mean (SD)		7.4 (2.4)	7.3 (2.4)	0.987 (0.938–1.039)	*P* = 0.615		
AO frag^a^	Mean (SD)		64.9 (14.6)	65.4 (14.3)	1.002 (0.991–1.014)	*P* = 0.698		
Comet frag^a^	Mean (SD)		19.4 (9.5)	18.5 (9.6)	0.989 (0.975–1.003)	*P* = 0.139		
TUNEL frag^a^	Mean (SD)		11.8 (14.8)	12.4 (13.9)	1.003 (0.993–1.012)	*P* = 0.569		
SCD halo area^a^	Mean (SD)		16.5 (6.4)	18 (6.0)	1.041 (1.008–1.075)	*P* = 0.014*	1.04 (1.007–1.075)	*P* = 0.018*

	**bioch to clin preg**	MET	41 (13.2)	269 (86.8)				
			35 (12.4)	248 (87.6)	1.080 (0.667–1.757)	*P* = 0.755		
Allocation (%)		ICSI (n = 297)	40 (13.5)	257 (86.5)				
		PICSI (n = 296)	36 (12.2)	260 (87.8)	1.124 (0.694–1.826)	*P* = 0.634		
Female age^a^	Mean (SD)		3.3 (0.4)	3.3 (0.4)	0.951 (0.528–1.707)	*P* = 0.867		
Male age^a^	Mean (SD)		3.4 (0.5)	3.6 (0.5)	1.660 (1.039–2.706)	*P* = 0.038*		
HBS^a^	Mean (SD)		7.4 (2.5)	7.3 (2.4)	0.991 (0.882–1.104)	*P* = 0.880		
AO frag^a^	Mean (SD)		65.2 (14.1)	65.4 (14.4)	1.001 (0.977–1.025)	*P* = 0.921		
Comet frag^a^	Mean (SD)		16.2 (8.2)	18.8 (9.8)	1.031 (0.998–1.069)	*P* = 0.076		
TUNEL frag^a^	Mean (SD)		14.2 (14.3)	12.2 (13.8)	0.991 (0.972–1.012)	*P* = 0.347		
SCD halo area^a^	Mean (SD)		18.8 (4.4)	18.0 (6.1)	0.976 (0.895–1.056)	*P* = 0.561		

	**Live birth**	MET	47 (17.7)	219 (82.3)				
			45 (18.5)	198 (81.5)	0.944 (0.601–1.486)	*P* = 0.804		
Allocation (%)		ICSI (n = 253)	60 (23.7)	193 (76.3)				
		PICSI (n = 255)	32 (12.5)	225 (87.5)	2.186 (1.375–3.531)	*P* = 0.001**	2.167 (1.084–4.464)	*P* = 0.031*
Female age^a^	Mean (SD)		3.5 (0.4)	3.3 (0.4)	0.373 (0.205–0.664)	*P* = 0.001**	0.301 (0.113–0.761)	*P* = 0.013*
Male age^a^	Mean (SD)		3.7 (0.6)	3.5 (0.5)	0.677 (0.451–1.021)	*P* = 0.061		
HBS^a^	Mean (SD)		7.6 (2.1)	7.3 (2.5)	0.946 (0.844–1.051)	*P* = 0.319		
AO frag^a^	Mean (SD)		6.9 (1.1)	6.5 (1.5)	0.780 (0.601–0.997)	*P* = 0.054*	0.788 (0.602–1.016)	*P* = 0.073
Comet frag^a^	Mean (SD)		2.1 (1.0)	1.8 (1.0)	0.786 (0.603–1.029)	*P* = 0.076		
TUNEL frag^a^	Mean (SD)		10.2 (10.3)	12.6 (14.4)	1.014 (0.992–1.040)	*P* = 0.239		
SCD halo area^a^	Mean (SD)		17.9 (5.6)	18.0 (6.3)	1.003 (0.938–1.068)	*P* = 0.939		

Odds ratios (ORs) are shown for clinical outcome measures compared with patient baseline characteristics by univariable (Uv) or multivariable (Mv) regression. They are ordered according to gestational progression with fertilization rates leading to the formation of pronucleate zygotes (fert pnz) to biochemical pregnancy (biochem preg) following embryo transfer(s) indicated by detection of urinary hcGH, to conversion of a biochemical to a clinical pregnancy (bioch to clin preg), indicated by ultrasound and finally to live birth (liv brth). Sample sizes differ according to clinical progression with all clinical outcomes reported as a fraction of the full mechanistic cohort less two couples with no eggs (n* *=* *1245). Calculations are based on clinical outcomes at each gestational stage as indicated by 0 (negative) or 1 (positive). Hence embryo transfers were recorded for 1162 (93.3%) couples in the mechanistic cohort. Of these, 593 (51%) women were biochemically pregnant, 517 (41.5%) established a confirmed clinical pregnancy, 418 (35.6%) went on to a live birth and 92 miscarried. No treatment outcomes beyond clinical pregnancy were recorded for 8 couples. The models indicate that only fertilization and live birth rates differed significantly between the trial arms (following Mv regression). Values for all other baseline parameters also reflect all patients in the mechanistic cohort (Table II).

AO, acridine orange; HBS, hyaluronan binding score; MET, multiple embryo transfers; Mv, multivariable regression; SCD, sperm chromatin dispersion; TUNEL, terminal deoxynucleotidyl transferase dUTP nick end-labelling; Uv, univariable.

a Signifies data aggregated by decade interval for patient age or by 10-point difference for all other measurements.

Assays of DNAq (AO, Comet, SCD and TUNEL) reported as % sperm showing DNA fragmentation (frag except SCD which measures halo area in pixel^2^).

HBS reported as % motile sperm binding to the Hydak slide. * Indicates high significant (p < 0.05); ** Indicates very highly significant (p < 0.001).

At the other end of gestational progression, the marked decrease in live birth rates in the ICSI arm (Fig. 4) was strongly mitigated by PICSI for both advancing female (Fig. 4A) and male (Fig. 4B) age, although modelling suggested this effect was driven more strongly by female ageing. See also Supplementary Fig. S1 for the reciprocal fall in miscarriage rates following PICSI. The two assays of DNAq plotted include AO (Fig. 4C), retained in the model following multivariable regression and also Comet assay (Fig. 4D), which although parsimoniously dropped was weakly predictive by univariable regression and so shown here. The far narrower scatter surrounding the trends for PICSI compared with ICSI, particularly with AO data, is explained by the removal of patient ageing as a significant factor predicting live birth rates among the PICSI cohort. These figures also show that, regardless of treatment, a declining sperm DNAq was associated with a reduced predicted live birth rate.

Subsequent figures show the relationships between sperm HBS and DNAq for intermediate clinical outcomes. The model predicting the establishment of a biochemical pregnancy (Fig. 5) achieved in approximately half of all embryo transfers suggested a significantly deleterious effect of advancing male age (Fig. 5A) with a lesser effect of increasing female age (Fig. 5B) based on univariable regression. A significant increase in SCD halo scores, supporting biochemical pregnancy (Fig. 5C), was also evident. Male (Fig. 5A) but not female (Fig. 5B) age affected the modelled rates of subsequent conversion to a clinical pregnancy (Fig. 6) while none of the assays were predictive. Larger halo areas in SCD assays and lower frequencies of sperm with DNA damage assayed by AO and Comet indicate higher DNAq, reflecting more mature sperm chromatin compaction. Clinical effects of multiple embryo transfer (MET) were apparent in the jump in their proportion among the biochemically pregnant, from approximately 51% to 87% among the clinically pregnant falling to 82% of couples achieving a live birth outcome (Table IV). There were no differences in the numbers of METs between the PICSI and ICSI cohorts for all treatment outcomes.

### Relationship between mechanistic and full trial data

These findings prompted us to return to the full trial data, focusing on the establishment of clinical pregnancies and their outcomes (Supplementary Fig. S2). Here, female age was a clear indicator for establishing a clinical pregnancy (OR 0.66, 95% CI 0.56–0.77, *P* < 0.0001, per decade) continuing to live birth (OR 0.43, 95% CI 0.30–0.6024, <0.0001, per decade). The mitigating effect of PICSI on reducing the impact of ageing on live birth outcomes was also clear (OR 0.58, 95% CI 0.40–0.82, *P* = 0.002, per decade) and while there was no effect of PICSI on clinical pregnancy rates (OR 0.98, 95% CI 0.84–1.15, *P* = 0.80, per decade), the intervention clearly benefitted older women (≥35 years) (OR 0.41, 95% CI 0.25–0.68, *P* = 0.0006, per decade) more than younger (<35 years) women (OR 0.79, 95% CI 0.48–1.32, *P* = 0.371, per decade).

## Discussion

HA is an evolutionarily conserved, ancient constituent of the extra-cellular matrix found throughout nature (Kogan *et al.*, 2007). In animals, HA forms complex structural matrices and substrates for adhesion by and motility of many cell types, including sperm, through cell-surface HA receptors, several of which have been described (Pilarski *et al.*, 1994; Martin-Deleon, 2011; Zhu *et al.* 2013; Torabi *et al.*, 2017). HA-enriched ‘glues’ are commonly used in IVF settings to affix embryos to plastic substrates and a similar principle is applied to the immobilization and capture of sperm for ICSI (Yagci *et al.*, 2010; McDowell *et al.*, 2014), including solid-state PICSI. The development of HA-based sperm selection processes was justified on the grounds that HA-binding sperm are demonstrably more mature, have higher motility and better indices of good DNAq (Yagci *et al.*, 2010; Torabi *et al.*, 2017). The commercially available variant of PICSI used in HABSelect, is a solid-state HA-binding platform developed originally by Biocoat USA using their Hydak process, also used in the scoring of slides to obtain HBS values. The more closely controlled production process of solid-state PICSI aided its consistent performance across the multiple sites participating in the associated RCT. One small RCT comparing PICSI with SpermSlow suggested they are equivalent and may be considered interchangeable, although we were not in a position to confirm this (Parmegiani *et al.*, 2012).

PICSI was only used to prospectively select sperm for clinical treatment (Miller *et al.*, 2019). Importantly, all fresh samples used for assessment of DNAq were *residual* to treatment and not separated into HA-selected versus unselected sperm beforehand. HBS was always obtained *before* sample processing and freezing. As both HBS and DNAq data were considered retrospectively and post-randomization, neither had a bearing on either patient management or treatment outcomes. Despite this temporal disconnection between them, relationships between HBS, DNAq and sperm baseline physiological measures were preserved and hence comparable with each other.

HBS has been reported previously to correspond with standard measures of semen quality and associated clinical outcomes, with an arbitrary value of ≤65% binding indicating a less fertile ejaculate (Huszar *et al.*, 2003; Tarozzi *et al.*, 2009; Mokanszki *et al.*, 2014; Rashki Ghaleno *et al.*, 2016; Erberelli *et al.*, 2017). Prior clinical trials of PICSI have used the ≤65% value in their inclusion criteria (Worrilow *et al.*, 2012; Mokanszki *et al.*, 2014). We did not set or apply thresholds or other cut-offs when reporting measures of HBS or DNAq. We looked instead for trends in all assay measures of sperm quality according to physiological patient baseline data and to clinical treatment outcomes. All measures and trial outcome data were integrated with the aim of exploring relationships between them and, in turn, generating explanatory hypotheses. The statistician responsible for selecting samples for the mechanistic analyses reported here (R.W.) ensured throughout that mechanistic laboratories remained blind to the associated treatment arm and their respective clinical outcomes. Males included in the HABSelect trial had relatively relaxed inclusion and exclusion criteria (Witt, 2016). Essentially only the relatively small numbers of men who could not provide a fresh sample on the day of treatment or had undergone treatment for cancer in the previous 24 months or a vasovasotomy procedure were excluded. Hence, semen samples displayed a wide range of phenotypes from normozoospermic to severely oligozoospermic. Following the WHO 2010 lower reference values for sperm concentration and progressive motility (Cooper *et al.*, 2010), twice as many abnormal as normal samples were found in the full trial cohort and in the mechanistic cohort. As randomization would have equalized the proportions of these samples in both arms of the trial, this 2:1 ratio was preserved throughout. Any effects on embryo quality and clinical outcomes influenced by treatment could only have arisen, therefore, via some feature(s) common to sperm in both arms, but sensitive to the PICSI intervention.

In this regard, only two effects were observed. The first was the significantly larger number of injected eggs required to obtain similar numbers of PNZs in the PICSI as in the ICSI arms (8.94 eggs for 6.02 PNZs on average compared with 8.76 eggs for 6.22 PNZs), a reduction that may have been physical and/or physiological in nature. Some aspect of PICSI could have led to the selection of sperm with lower levels of the egg-activating factor, phospholipase C _ζ_ (Swann and Lai, 2016), for example. Alternatively, repeated attempts to detach strongly bound sperm from the HA substrate may have damaged sperm membranes and as polyvinylpyrrolidone (PVP) was routinely used to hold sperm prior to ICSI, regardless of treatment allocation, potentially toxic effects of the chemical (Kato and Nagao, 2009) may have become more apparent following PICSI. Inevitably longer delays between sperm selection by PICSI and injection, as reported by numerous clinics (personal communication), may also have been a factor. Because they were reported on a per treatment cycle basis rather than by total number of eggs injected as reported here, fertilization rates in the original HABSelect report did not differ significantly between treatment arms (Miller *et al.*, 2019). Other reports have indicated either no differences or higher rates of fertilization and other outcomes with PICSI-selected sperm, although these reports had considerably smaller cohorts than HABSelect (Parmegiani *et al.*, 2010; Majumdar and Majumdar, 2013; Mokanszki *et al.*, 2014; Novoselsky Persky *et al.*, 2021). Differences in fertilization rates had no bearing on subsequent treatment outcomes. The second and more clinically relevant effect was the mitigation in declining live birth rates among older couples following PICSI, particularly those with an older female partner (HFEA, 2016). As this effect was only apparent at a comparatively later stage in the gestational progression and in older women, the underlying ‘defect’ most likely involved both male and female contributions.

There is abundant evidence in the literature that poor sperm DNAq is frequently incompatible with successful reproductive outcomes for both standard IVF and ICSI (Robinson *et al.*, 2012; Simon *et al.*, 2014; Zhao *et al.*, 2014; Bach and Schlegel, 2016; Cissen *et al.*, 2016; Simon *et al.*, 2017), with evidence suggesting that one of the main effects of ICSI manifests through a higher risk of miscarriage (Worrilow *et al.*, 2012; Mokanszki *et al.*, 2014; Erberelli *et al.*, 2017). While any DNA damage introduced by the sperm is likely to be genotoxic, the chances of the zygote tolerating and recovering from the damage would depend on the type and extent of the damage encountered. In the context of the HABSelect study, the Comet, TUNEL and AO assays all focused on detecting single and/or double-stranded SDF, which arises as the protective effect of incomplete chromatin compaction falls (Agarwal *et al.*, 2016). As it measures a regressive loss of torsional stress reflecting poor chromatin compaction and correspondingly elevated DNA fragmentation, the SCD-based halo assay lies somewhere between the two (Fernandez *et al.*, 2003). This may explain why, in HABSelect, scores obtained by SCD aligned more closely with reduced pregnancy rates, possibly arising from fundamental DNA packaging errors in the fertilizing sperm (Hammadeh *et al.*, 2001; Kim *et al.*, 2013), while scores obtained by AO and Comet assays aligned more closely with later failures in the maintenance of pregnancy, possibly arising from DNA damage. Rather than fundamental irreparable packaging errors that would most likely cause fertilization or very early gestational failures (Oliva, 2006; Nasr-Esfahani *et al.*, 2008; Castillo *et al.*, 2011), we think our evidence points to repairable DNA stand-breaks in the fertilizing sperm being the most likely male factor responsible for falling live birth rates among older women that are rescued by PICSI. A proposed model tying this hypothesis into the ageing female germ line is further outlined below.

Evidence is also accumulating that sperm DNAq decreases with rising male age (Deenadayal Mettler *et al.*, 2020; Vaughan *et al.*, 2020; Gao *et al.*, 2021). However, despite the significant differences in all measures of DNAq between normal and abnormal samples in the HABSelect study, there was no significant difference in the age range of the men providing the samples (although a trend for falling fertilization rates among couples with older male partners in the ICSI but not the PICSI cohort was noted). Older men produced samples with higher concentrations of sperm (data not shown), a phenomenon reported elsewhere in a study showing corresponding decreases in sperm DNAq (Deenadayal Mettler *et al.*, 2020). While male and female ages were highly correlated in HABSelect, the model for predicting biochemical pregnancy retained only male age as a significantly associated variable and this effect was also apparent for conversion to clinical pregnancy, albeit more weakly. Hence, in the HABselect mechanistic cohort, male age may have had more of an impact on gestational progression from fertilization through to implantation, while female age was retained as a significant variable in the model predicting live birth/miscarriage outcomes, essentially agreeing with accepted trends for women undergoing fertility treatment (Cimadomo *et al.*, 2018; Ubaldi *et al.*, 2019). These studies have been unable to conclude if the observed deterioration in sperm quality with ageing impacted on ART outcomes; by demonstrating, however, that all models predicting clinical outcomes retained either some aspect of sperm DNAq or male age, HABSelect’s mechanistic analysis suggests that sperm DNA lesions, possibly of differing qualities, were impacting all stages of gestation and may have had immediate or more delayed impacts on developmental progression, best measured (in our hands) respectively, by SCD or by AO and Comet. TUNEL was not retained in any of the models predicting clinical outcomes.

Esteves *et al.* (2021) suggested a proposed categorization of DNAq assays based on their modus operandi (described in some detail by Agarwal *et al.* (2016)). They grouped SCD with *in situ* nick translation, the sperm chromatin structure assay (SCSA), the Comet and TUNEL assays into a broad category for measures of SDF. They also grouped AO and Aniline Blue (AB) together with chromomyacin A3 (CMA3) and toluidine blue into a broad category for measures of chromatin compaction. As we argue above, relative chromatin compaction is closely associated with differential levels of DNA fragmentation, hence these categories, in our view, are not mutually exclusive. Categorization based on whether an assay is considered a direct (AO Comet and TUNEL) or indirect (AB, CMA3, SCD) measure of DNAq may be more relevant (Cho *et al.*, 2017; Ribas-Maynou, 2021). SCD was retained by our model examining earlier outcomes and AO and Comet by models examining later outcomes. These relationships were relatively weak, however, and may have been coincidental.

In relation to clinical outcomes, distinguishing between reports based on processed (enriched, normally for better quality as in HABSelect) and unprocessed (mixed) populations of sperm is not straightforward (Zini, 2011; Simon *et al.*, 2017). The meta-analysis of Cissen *et al.* (2016) of 30 studies that included SCSA, TUNEL, SCD and Comet assays, reported a poor prediction for clinical pregnancy after IVF or ICSI regardless of how sperm were processed. An earlier report (Collins *et al.*, 2008) drew a similar conclusion with TUNEL and SCSA assays where pelleted populations were enriched for better quality sperm beforehand. Enrichment is essentially the premise behind the proposed ‘iceberg’ effect, defined as the underestimation of sperm with poor DNAq because of their prior elimination by sample processing (Alvarez and Lewis, 2008; Gosalvez *et al.*, 2013). This effect may also have a bearing on the increased risk of miscarriage in ICSI cycles associated with using unprocessed semen (Robinson *et al.*, 2012; Zhao *et al.*, 2014; Coughlan *et al.*, 2015; Cissen *et al.*, 2016).

Despite the importance of appropriate sperm DNA condensation for successful fertilization and early development (Schlicker *et al.*, 1994; Nili *et al.*, 2009; Ovari *et al.*, 2010), PICSI had no significant impact on biochemical or clinical pregnancy rates. Hence, the avoidance of fundamental sperm DNA packaging errors (Schlicker *et al.*, 1994; Nili *et al.*, 2009; Francis *et al.*, 2014; Hamidi *et al.*, 2015; Asmarinah *et al.*, 2016) was unlikely to be as relevant to increased miscarriage risk mitigated by PICSI as DNA strand breaks and/or associated oxidative lesions in DNA involving adducts (De Iuliis *et al.*, 2009). Tying in the female factor to the PICSI mitigation of ageing on reduced live birth rates, we think that while the human oocyte can probably tolerate and repair a certain level of sperm DNA damage, tolerance progressively diminishes as the oocytes’ biological age rises (Nunez-Calonge *et al.*, 2012; Perry, 2015; Fernandez-Diez *et al.*, 2016; Ribas-Maynou and Benet, 2019; Horta *et al.*, 2020). By selecting sperm with lower levels of DNA damage in the PICSI arm of the trial, it is likely that the DNA repair machinery of biologically older oocytes had less demand placed on it and hence their lower tolerance thresholds (to DNA damage) were less frequently breached. We think this explanation applies to the study of Worrilow *et al.* (2012), which reported the mitigation of miscarriage in their PICSI cohort, although they did not consider whether this was age-related. The retention of at least one DNAq assay in the model of fertilization (negatively affected by PICSI) could also be explained by mechanical disruption that may have activated sperm caspases, triggering an apoptotic cascade with DNA strand breaks and ultimately, fertilization failure (Cayli *et al.*, 2004; Sakkas and Alvarez, 2010; Aitken and Koppers, 2011). The retention of the Comet assay by this model likely reflects sample processing and technical considerations rather than any unique biological factor.

On a cautionary note, high levels of variability (noise) in our DNAq data made direct binary comparisons (e.g. between predicted rates of biochemical pregnancy and the Comet assay) uninformative. The origin of the noise lay in the sampling itself. The necessary multi-centre approach to sample acquisition and processing was one important source. Moreover, processed samples were normally ‘cleared’ of much of the poorer quality sperm that failed to penetrate the 80–90% gradient layers or swim up effectively enough (Cooper *et al.*, 2010; Jackson *et al.*, 2010; Gosalvez *et al.*, 2011; Torabi *et al.*, 2017). Hence, while sample processing improved quality for clinical treatment, it also removed potentially useful ‘signals’ (the aforementioned ‘iceberg’ effect) for subsequent mechanistic analysis. Moreover, our use of clinically approved freezing protocols designed for raw semen on processed samples may have introduced noise through iatrogenic effects. Paradoxically, these effects may also have helped reveal differences in sperm DNAq as measured by different assays that had differential effects on clinical outcomes (Amir *et al.*, 2019). Quid pro quo, the sperm used for treatment and for the mechanistic analysis in the HABSelect study came from the same processed samples and so are directly comparable.

As ICSI-based treatments, regardless of need, continue to rise as a proportion of all treatment cycles (Dyer *et al.*, 2016) alternative methods, including HA-binding, are being developed for enriching sperm of a higher quality for use in ICSI procedures (Lepine *et al.*, 2019). Based on our models’ outputs, extending on the findings of our original report (Miller *et al.*, 2019), we hypothesize that the reduction in miscarriage in the trial’s PICSI arm was linked to the more successful avoidance of sperm with a repairable defect in their DNA. The defect did not necessarily prevent progression to clinical pregnancy but once established, failed to maintain it, mainly among older women. Samples refractory to the PICSI mitigation of miscarriage were likely caused by factors that were not restricted to sperm or, if carried by sperm, caused earlier treatment failures. We could not check for the avoidance of aneuploid sperm by PICSI, but HA-selected sperm have previously been reported to have lower frequencies of aneuploidies (Cayli *et al.*, 2003; Huszar *et al.*, 2006) and if present, these would more likely have caused earlier treatment failures (Jenderny, 2014). Moreover, as trisomies originate mainly in the female germ line, they were unlikely to be responsible for the male factor mitigated by PICSI in HABSelect. Our analysis demonstrating the clear relationships between sperm physiological parameters, HBS and DNAq suggests that the male factor mitigated by PICSI was an aspect of DNAq associated with or causing a subtle deficiency in sperm phenotype, including HA-binding capacity (Huszar *et al.*, 2003; Cayli *et al.*, 2004; Prinosilova *et al.*, 2009).

To conclude, although it was argued at the time that mitigation of miscarriage risk by PICSI as reported in our original study could have been a chance finding, our mechanistic analysis suggests otherwise. A reduction in rates of miscarriage is the one consistent feature of HA-selection shared with the only other large clinical trial of the (PICSI) intervention to date (Worrilow *et al.*, 2012) and in several smaller studies (Majumdar and Majumdar, 2013; Mokanszki *et al.*, 2014). Alongside the clear relationship with patient ageing confirmed by modelling in this follow-up study, our evidence points to the effect being bona fide and that a male factor, most likely a genotoxic sperm DNA defect, may be responsible for up to one-third of miscarriages. No other detail of the data, such as METs or differences in embryo quality, offers an alternative explanation. Confirmatory RCTs with older couples and/or couples with abnormal semen samples should now be designed to consolidate this finding alongside the evaluation of different versions of PICSI, including liquid-state options, which could additionally substitute for PVP. Furthermore, a future mechanistic study should focus on differences in DNAq between HA-binding and non-binding sperm to more clearly identify the factor(s) that the selection helps avoid.

## Data availability

Data available on request. The data underlying this article will be shared on reasonable request to the first or corresponding authors.

## Supplementary Material

deac058_Supplementary_data

deac058_Supplementary_Figure_S1

deac058_Supplementary_Figure_S2

deac058_Supplementary_Table_SI

deac058_Supplementary_Table_SII

deac058_Supplementary_Table_SIII
