# Supplementary material for: Sperm selection with hyaluronic acid improved live birth outcomes among older couples and was connected to sperm DNA quality, potentially affecting all treatment outcomes
Source: Hum Reprod. 2022 Apr 23;37(6):1106–25. doi: 10.1093/humrep/deac058 (PMC9156852; doi:10.1093/humrep/deac058)
Supplement: deac058_Supplementary_data [file deac058_supplementary_data.pdf]

Human Reproduction, pp. 1–2, 2022
https://doi.org/10.1093/humrep/deac058

Supplementary data

Semi-automated acquisition of acridine orange (AO) scores

Acridine orange (AO) is a metachromatic fluorescence stain whose intensity and colour indicate the presence of double-stranded (green) or single-stranded (orange/red) state, of DNA (or RNA), respectively. In the context of this work, the assay measured an aspect of DNA fragmentation in sperm nuclei. A subjective manual assessment was made for audit purposes, followed by an objective automated analysis for high throughput classification. This phenotype classifier had a minimal scope of >50 individual spermatozoa within each field of view.

Staining

All treated semen samples were delivered frozen in 250 µl aliquots and stored at −80°C prior to processing. On thawing, spermatozoa were washed out of cryoprotectant using neutral Tris buffered saline and for AO staining were spread on poly-L-lysine coated slides and allowed to dry. The protocol published by Yagci *et al.* (2010) was followed throughout. Briefly, slides were sequentially treated (30 s each) with solutions of 0.1 M HCl followed by 0.1 M NaOH. Sperm were then fixed in modified Carnoy's solution (9:1) methanol:acetic acid for 5 min at room temp. Slides were flooded with a solution of freshly prepared acridine orange (Polysciences Inc, UK, final conc 12 μg/ml) under orange safe light for 5 min at room temperature before decanting of reagent and washing slides with distilled water (×3–×5 times) and allowing to air dry. Slides were finally mounted in DPX solution prior to visualization and kept in the dark for storage and imaging. Please see main manuscript for full description of staining protocol.

Image acquisition and processing

AO fluorescence images were acquired from a Zeiss Axiomat microscope fitted with epifluorescence optics for similar blue excitation (494 nm) and specific characteristics for green (530 nm) and orange (600 nm) fluorescence at 400× final magnification. Images were recorded on a cooled scientific 16-bit greyscale camera (*Hamamatsu*) with SmartCapture software on macOS (10.11). As manual imaging was impracticable, we chose to set the (SmartCapture) software for automatic exposure setting and recording and use of these settings to correct for over or under exposure of all captured fields. A custom pipeline in *ImageJ* software (Fiji distribution) was written for this purpose, independently measuring the 16 bit greyscale levels stored in the two-channel image comprising orange- and green-emission filtered exposures of the same field. The typical image size was 1300 × 1000 pixels, at 40× magnification in a field of 250 × 200 microns. The first step used an AppleScript macro (supplied on request) converting nested folders of SmartCapture 3 Filmstrip files to two-channel TIFFs. The macro also collated the exposure times for each channel to a text file. A second Applescript file (supplied on request) ensured conformity in the filename syntax. A traditional image-processing pipeline, as described below, was written as an ImageJ macro file (supplied on request) targeting nested folders of SmartCapture3 TIFF files as input. This carried out pre-processing and segmentation steps for each image and then calculated and stored the mean and SD of the pixel values for each derived sperm segment in both green and orange frames.

For pre-processing, nested folders for input images were selected with a specific syntax based on SmartCapture TIFF export. The background is first subtracted using a rolling-ball algorithm (radius 50 pixels or 8 µm) to tare the 'zero' pixel value to foreground contributions only. Individual sperm nuclei are then segmented so that measurements can be recorded for individual sperm. Taking a maximum-value projection of the image and thresholding above some constant pixel brightness (220/255 in 8-bit, upper 14% of range) gives a binary mask from only bright foreground objects, excluding less than 0.5% of sperm cells. The closed islands in the mask are then split into individual cells by watershed algorithm. To eliminate debris and non-sperm cells, the resulting set of objects is filtered by area (50–1000 sq. pixels, or 1.3–26 μm^2) and circularity ($4\pi \times$ area/perimeter = 0.60–0.96).

For the uncorrected 'acquired colour ratio' (ACR), the mean and SD of the distribution of pixel values within each remaining object are calculated for orange and green frames. The ratio of the mean pixel values then forms a green/orange ACR for each nucleus.

$$\mathrm{ACR} = \frac{\text{mean green pixel value}}{\text{mean orange pixel value}}$$

As the uncorrected ACR is confounded by varying exposure times for green and orange, it is skewed by the relative brightness of sperm cells to other objects in the same field and therefore does not represent the objective colour profile of the sperm. Since the actual light intensity emitted from each object is proportional to the rate at which pixel brightness accumulates over an exposure; that is to say, the mean pixel value divided by the corresponding exposure time, a corrected 'intensity colour ratio; ICR' of the true intensities in the two wavebands can be calculated as follows

$$\begin{aligned}\mathrm{ICR} &= \frac{\text{mean green pixel value/green exposure time}}{\text{mean orange pixel value/orange exposure time}} \\ &= \mathrm{ACR}\ C\frac{\textit{orange exposure time}}{\textit{green exposure time}}\end{aligned}$$

SmartCapture records the exposure times automatically at the point of acquisition. The correction factor is then applied for both fixed and automatic exposure settings. For less damaged (green) sperm, ICR >1, and ICR <1 for an orange sperm. For each sample, the ICR distributions over multiple sperm images have a mean and standard

deviation reported as is. For comparison with other measures of DNA fragmentation, the ICR is converted to an effective DNA fragmentation index ranging from 0% (entirely green, no orange above background) to a maximum of 100% (entirely orange, no green above background). We define this as:

$$\mathrm{DFI} = \frac{100\%}{1 + \left(\frac{\text{mean green pixel value}}{\text{mean orange pixel value}} \times \frac{\text{orange exposure time}}{\text{green exposure time}}\right)} = \frac{100\%}{1 + ICR}$$

To export corrected image acquisition data, a Python script (Supplementary AO4: AOexcelcollator_TIFFexposures.py) collated the ImageJ results and the exposure times from the nested sample folders to a spreadsheet, in which the ICR and DFI were calculated for each sperm.

Since the result for each sperm lies on a quantitative, interval scale, it is not necessary to bin the fraction of cells whose DFI lies above an arbitrary threshold (e.g. 50%) to generate a per-sample metric. Instead, the mean and standard deviations of the per-sperm ICR and DFI metrics were calculated and reported for each sample containing sufficient detected sperm ($n > 50$).

References

Yagci A, Murk W, Stronk J, Huszar G. Spermatozoa bound to solid state hyaluronic acid show chromatin structure with high DNA chain integrity: an acridine orange fluorescence study. *J Androl* 2010;**31**:566–572.
