# Supplementary material for: Sperm selection with hyaluronic acid improved live birth outcomes among older couples and was connected to sperm DNA quality, potentially affecting all treatment outcomes
Source: Hum Reprod. 2022 Apr 23;37(6):1106–25. doi: 10.1093/humrep/deac058 (PMC9156852; doi:10.1093/humrep/deac058)
Supplement: deac058_Supplementary_Figure_S1 [file deac058_supplementary_figure_s1.pdf]

Human Reproduction, pp. 1–1, 2022
https://doi.org/10.1093/humrep/deac058

human reproduction

SUPPLEMENTARY DATA

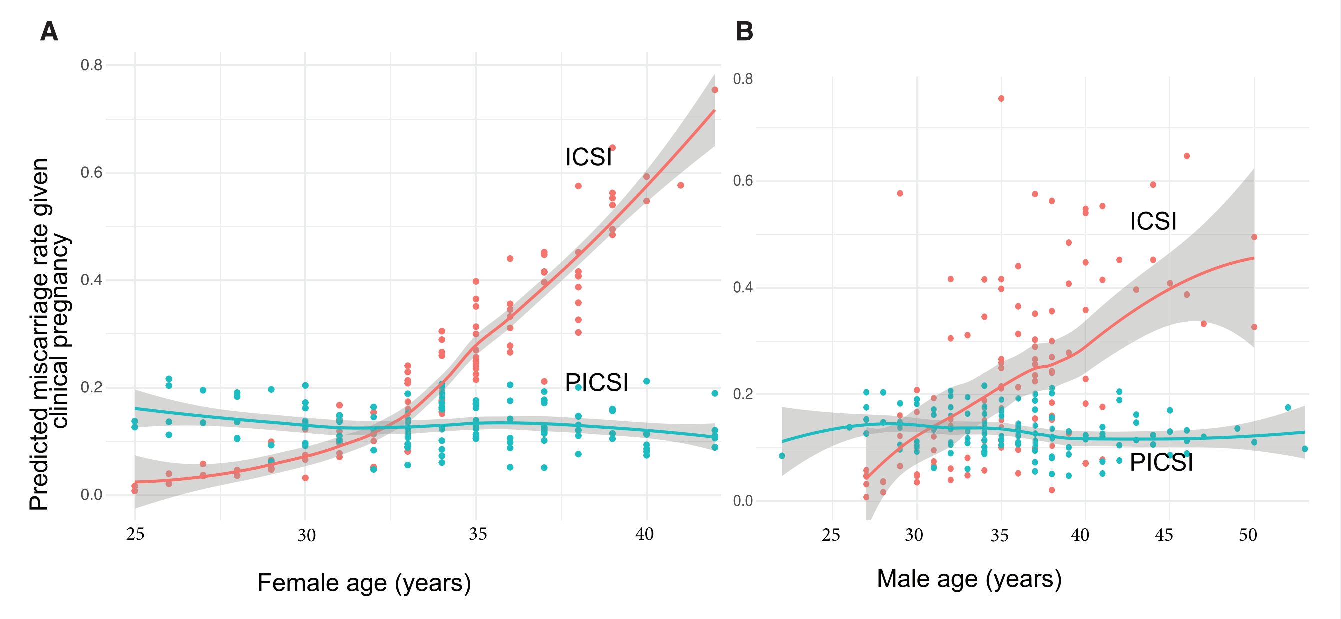

Supplementary Figure S1 Predicting miscarriage rate following ICSI or PICSI. The plots are the inverse relationships shown in Fig. 4 for female (**A**) and male (**B**) age. Scales for miscarriage rates are shown ranging from 0% (0.00) to 80% (0.80). PICSI, physiological intracytoplasmic sperm injection.
