# Supplementary material for: Sperm selection with hyaluronic acid improved live birth outcomes among older couples and was connected to sperm DNA quality, potentially affecting all treatment outcomes
Source: Hum Reprod. 2022 Apr 23;37(6):1106–25. doi: 10.1093/humrep/deac058 (PMC9156852; doi:10.1093/humrep/deac058)
Supplement: deac058_Supplementary_Figure_S2 [file deac058_supplementary_figure_s2.pdf]

Human Reproduction, pp. 1–1, 2022
https://doi.org/10.1093/humrep/deac058

human reproduction

SUPPLEMENTARY DATA

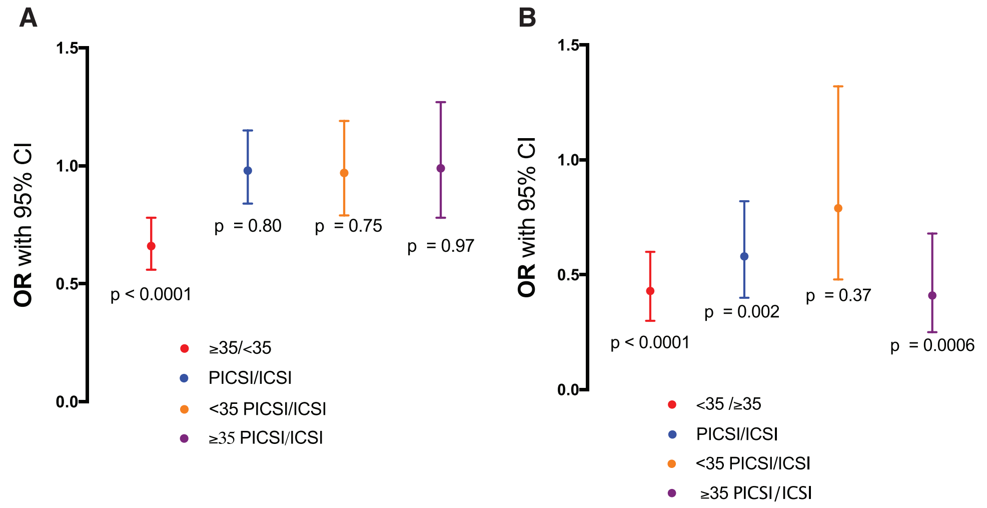

Supplementary Figure S2 ***Post hoc*** **analysis of outcomes for the full trial cohort.** Following the mechanistic analysis, we returned to the full trial cohort seeking confirmation of the impact of both age and treatment allocation on the odds of establishing a clinical pregnancy (**A**) and the subsequent odds of miscarriage (**B**). Only (female) age was a significant factor in the establishment of clinical pregnancy and there was no effect of treatment allocation. In contrast, both age and treatment allocation had strong effects on the odds of miscarriage, with our analysis clearly indicating that older women benefitted most from PICSI. Sample sizes for clinical pregnancy (CP) versus not pregnant (NP) are NP <35 years: ICSI/PCSI, 455/466; ≥35 years: 429/429 and CP <35 years: 304/301; ≥35 years: 187/186. Sample sizes for live birth (LB) versus miscarriage (MC) are MC <35 years: ICSI/PCSI, 38/31; ≥35 years: 58/29 and LB <35 years: 262/269; ≥35 years: 129/156. PICSI, physiological intracytoplasmic sperm injection.
