# Supplementary material for: Sperm selection with hyaluronic acid improved live birth outcomes among older couples and was connected to sperm DNA quality, potentially affecting all treatment outcomes
Source: Hum Reprod. 2022 Apr 23;37(6):1106–25. doi: 10.1093/humrep/deac058 (PMC9156852; doi:10.1093/humrep/deac058)
Supplement: deac058_Supplementary_Table_SI [file deac058_supplementary_table_si.pdf]

Human Reproduction, pp. 1–1, 2022
https://doi.org/10.1093/humrep/deac058

human reproduction

SUPPLEMENTARY DATA

Supplementary Table SI **Full data for violin plots shown in Fig. 2.**

Assay	Min		1stQ		Median		Mean		3rdQ		Max		*P*-value
	N	A	N	A	N	A	N	A	N	A	N	A	
**AO**	20.96	21.19	54.92	59.22	63.01	66.59	63.87	66.55	74.79	75.87	95.51	92.83	0.026*
**Comet**	3.45	2.25	10.23	12.69	15.97	18.28	16.97	20.91	21.47	25.64	50.85	54.85	<0.001**
**TUNEL**	0.10	0.15	1.55	2.47	9.22	8.91	9.22	14,15	12.56	19.40	77.84	100.00	<0.001**
**SCD**	21.37	30.64	146.55	132.49	196.37	166.60	179.84	166.13	224.61	212.05	347.06	311.49	0.025*
**HBS**	3.50	1.00	74.75	55.00	87.50	81.00	79.88	70.43	93.00	90.75	99.50	100.00	<0.001**

All values are for % sperm with DNA fragmentation except for SCD, which is reported as halo area in $pixel^2$ and HBS which is % sperm binding to hyaluronic acid. *P*-values are reported following Mann–Whitney *U* test. Full details for scores and definitions thereof are given in Materials and methods and in the Supplementary Materials and Methods for AO.
A, abnormal classifications; AO, acridine orange; HBS, hyaluronan binding score; N, normal; SCD, sperm chromatin dispersion; TUNEL, terminal deoxynucleotidyl transferase dUTP nick end-labelling.
HBS reported as % motile sperm binding to the Hydak slide.
