# Supplementary material for: Sperm selection with hyaluronic acid improved live birth outcomes among older couples and was connected to sperm DNA quality, potentially affecting all treatment outcomes
Source: Hum Reprod. 2022 Apr 23;37(6):1106–25. doi: 10.1093/humrep/deac058 (PMC9156852; doi:10.1093/humrep/deac058)
Supplement: deac058_Supplementary_Table_SII [file deac058_supplementary_table_sii.pdf]

Human Reproduction, pp. 1–1, 2022
https://doi.org/10.1093/humrep/deac058

human reproduction

SUPPLEMENTARY DATA

Supplementary Table SII **Missingness in HABSelect inter-assay coverage.**

Inter-assay coverage (n)	Comet	TUNEL	AO	SCD (halo)	HBS	Assay missing?
195	1	1	1	1	1	0
230	1	1	1	0	1	1
132	1	1	0	1	1	1
143	1	1	0	0	1	2
20	1	0	1	1	1	1
46	1	0	1	0	1	2
17	1	0	0	1	1	2
57	1	0	0	0	1	3
20	0	1	1	1	1	1
22	0	1	1	0	1	2
13	0	1	0	1	1	2
43	0	1	0	0	1	3
3	0	0	1	1	1	2
9	0	0	1	0	1	3
1	0	0	0	1	1	3
86	0	0	0	0	1	4
8	1	1	1	1	0	1
12	1	1	1	0	0	2
3	1	1	0	1	0	2
8	1	1	0	0	0	3
1	1	0	1	1	0	2
5	1	0	1	0	0	3
1	1	0	0	1	0	3
9	1	0	0	0	0	4
6	0	1	1	1	0	2
3	0	1	1	0	0	3
3	0	1	0	1	0	3
29	0	1	0	0	0	4
2	0	0	1	0	0	4
2	0	0	0	1	0	4
86	0	0	0	0	0	5
**1215**	**328**	**345**	**633**	**790**	**178**	**2274**

Missingness in HABSelect inter-assay coverage.
There was variable coverage by the 5 assays of sperm quality among a total of 1215 samples considered in the mechanistic analysis. The table's first column breaks the total down by inter-assay coverage depending on whether a sample was (1) or was not (0) covered by the relevant assay as indicated in the columns to the right. For example, 195 samples had full coverage with no missing values and 86 samples had no coverage (all missing). Values on the bottom row in bold (excluding 1215) are the total numbers of samples without coverage for each of the assays. A grand total of 3801 assay values were obtained within the mechanistic dataset, corresponding to 62.5% coverage overall.
AO, acridine orange; HBS, hyaluronan binding score; SCD, sperm chromatin dispersion; TUNEL, terminal deoxynucleotidyl transferase dUTP nick end-labelling.
