# Supplementary material for: Sperm selection with hyaluronic acid improved live birth outcomes among older couples and was connected to sperm DNA quality, potentially affecting all treatment outcomes
Source: Hum Reprod. 2022 Apr 23;37(6):1106–25. doi: 10.1093/humrep/deac058 (PMC9156852; doi:10.1093/humrep/deac058)
Supplement: deac058_Supplementary_Table_SIII [file deac058_supplementary_table_siii.pdf]

Human Reproduction, pp. 1–1, 2022
https://doi.org/10.1093/humrep/deac058

human reproduction

SUPPLEMENTARY DATA

Supplementary Table SIII **Embryo quality and potential cumulative pregnancy rates.**

**Baseline data**<br>**n**		**Trans or frozen**<br>**501**	**Degen**<br>**527**	***P*-value**
Allocation = PICSI (%)		240 (47.9)	278 (52.8)	0.136
Abnormal samples (%)		338 (67.5)	339 (64.3)	0.32
Mean sperm conc mml (mean (SD))		25.83 (36.52)	26.22 (35.12)	0.863
% Prog for mot (mean (SD))		40.20 (19.82)	41.90 (18.81)	0.170
% HBS (mean (SD))		71.93 (26.11)	75.32 (23.07)	0.043*
AO frag (mean (SD))		66.87 (12.58)	64.72 (15.36)	0.095
Comet frag (mean (SD))		19.22 (9.63)	18.95 (9.38)	0.705
SCD halo area (mean (SD))		175.72 (63.97)	176.93 (63.22)	0.862
TUNEL frag (mean (SD))		12.85 (16.33)	12.74 (15.67)	0.783
**Embryo quality**				
Day 3 Cell Nr (median (IQR))		8 [6, 8]	7 [4, 8]	<0.001**
Day 3 Frag (median (IQR))		3 [3, 4]	3 [2, 4]	<0.001**
Day 5 Exp (median (IQR))		4 [2, 5]	4 [2, 7]	<0.005*
**All embryos (%)**		**Cryo** (%)	**Trans** (%)	**Degen** (%)
ICSI	7680 (50.2)	1387 (18.05)	1799 (23.41)	4494 (58.48)
PICSI	7605 (49.8)	1276 (16.78)	1761 (23.15)	4568 (60.06)

Numbers of transferred, frozen or discarded embryos with full sperm assay data including HBS in the mechanistic cohort (n = 1028) were considered and potential differences between category values were checked using *t* tests or Mann–Whitney *U* tests for differences, respectively, between means or medians. As expected for a population of samples randomized equally into either treatment arm (Baseline data), there were no differences in basic semen parameters or DNAq assays, although HBS was elevated among samples associated with degenerate embryos. Statistical support for the clinical decision of which embryo(s) to transfer or freeze, indicating higher quality embryos is shown (Embryo quality) where cell number at Day 3, expansion at Day 5 and fragmentation index at Day 3 were all significantly different compared with degenerate embryos. For the full trial cohort (All embryos; n = 2766), similar numbers of embryos were transferred in both arms. A slight reduction in the number of cryopreserved and a corresponding increase in the number of degenerate embryos were noted in the PICSI arm (neither statistically significant). These data indicate equivalence in the potential cumulative pregnancy rates regardless of treatment allocation.
AO, acridine orange; HBS, hyaluronan binding score; SCD, sperm chromatin dispersion; TUNEL, terminal deoxynucleotidyl transferase dUTP nick end-labelling.
